# Acute Beneficial Hemodynamic Effects of a Novel 3D-Echocardiographic Optimization Protocol in Cardiac Resynchronization Therapy

**DOI:** 10.1371/journal.pone.0030964

**Published:** 2012-02-03

**Authors:** Carolin Sonne, Lorenz Bott-Flügel, Simon Hauck, Hasema Lesevic, Petra Barthel, Fabian Michalk, Katharina Hoppe, Jörg Hausleiter, Albert Schömig, Christof Kolb

**Affiliations:** 1 Klinik für Herz- und Kreislauferkrankungen des Erwachsenen, Deutsches Herzzentrum München, Technische Universität München, Faculty of Medicine, Munich, Germany; 2 Abteilung für Innere Medizin/Kardiologie, Kreiskrankenhaus Erding mit Klinik Dorfen, Erding, Germany; Cardiovascular Research Institute Maastricht - Maastricht University, The Netherlands

## Abstract

**Background:**

Post-implantation therapies to optimize cardiac resynchronization therapy (CRT) focus on adjustments of the atrio-ventricular (AV) delay and ventricular-to-ventricular (VV) interval. However, there is little consensus on how to achieve best resynchronization with these parameters. The aim of this study was to examine a novel combination of doppler echocardiography (DE) and three-dimensional echocardiography (3DE) for individualized optimization of device based AV delays and VV intervals compared to empiric programming.

**Methods:**

25 recipients of CRT (male: 56%, mean age: 67 years) were included in this study. Ejection fraction (EF), the primary outcome parameter, and left ventricular (LV) dimensions were evaluated by 3DE before CRT (baseline), after AV delay optimization while pacing the ventricles simultaneously (empiric VV interval programming) and after individualized VV interval optimization. For AV delay optimization aortic velocity time integral (AoVTI) was examined in eight different AV delays, and the AV delay with the highest AoVTI was programmed. For individualized VV interval optimization 3DE full-volume datasets of the left ventricle were obtained and analyzed to derive a systolic dyssynchrony index (SDI), calculated from the dispersion of time to minimal regional volume for all 16 LV segments. Consecutively, SDI was evaluated in six different VV intervals (including LV or right ventricular preactivation), and the VV interval with the lowest SDI was programmed (individualized optimization).

**Results:**

EF increased from baseline 23±7% to 30±8 (p<0.001) after AV delay optimization and to 32±8% (p<0.05) after individualized optimization with an associated decrease of end-systolic volume from a baseline of 138±60 ml to 115±42 ml (p<0.001). Moreover, individualized optimization significantly reduced SDI from a baseline of 14.3±5.5% to 6.1±2.6% (p<0.001).

**Conclusions:**

Compared with empiric programming of biventricular pacemakers, individualized echocardiographic optimization with the integration of 3-dimensional indices into the optimization protocol acutely improved LV systolic function and decreased ESV and can be used to select the optimal AV delay and VV interval in CRT.

## Introduction

Despite progress in the treatment of heart failure the five year mortality still remains over 50% [1]. About one third of patients with heart failure show a widened QRS complex (≥120 ms) as a sign of conduction system disease [2],[3]. Cardiac resynchronization therapy (CRT) has evolved as the treatment of choice for patients with symptomatic heart failure, left bundle branch block/QRS widening (≥120 ms) and severely reduced systolic left ventricular (LV) function despite optimal medical therapy. Large studies showed that CRT not only improves quality of life and LV systolic function [4], [5], [6] but also leads to a reduction in mortality [7]. Nevertheless up to one third of patients, so called non-responders, do not symptomatically respond to this therapy [8], [9], [10], [11]. The exact reasons for lack of response are still unclear, but inadequate lead placement, scar burden, and also device settings may contribute.

Several studies showed that increased scar burden, especially in the postero-lateral LV segments, the preferred region of the LV lead positioning, may lead to suboptimal clinical outcome [12], [13], [14]. This may be due to regional variations in electrical excitability and impulse propagation in proximity of the lead. Transvenous LV lead implantation is limited by the individual anatomy of the tributaries of the coronary sinus and sometimes by technical aspects concerning the attainability of the target vein. Thus, if one takes into account potential suboptimal LV lead placement, electrical latency during LV stimulation and slowed conduction due to scars near the LV pacing site, all possibly contributing to a reduced response to CRT, an individualized approach to programming CRT systems, with the possibility of pre-activation of either one of the ventricular leads, is intriguing.

In the clinical setting programming of CRT systems is frequently done empirically [15], using an AV delay of 120 ms and simultaneous biventricular pacing, without further optimization.

In small studies it has been shown that optimized programming of the AV delay leads to improved hemodynamics, as well as to improved symptomatic response and LV systolic function in the short and the longer term [16], [17],[18], [19], [20]. As with AV delay, acute hemodynamic benefits [21], [22], as well as symptomatic and echocardiographic advantages in the longer term [23], [24] have been described with interventricular VV interval optimization.

There are scarce studies that evaluated the effect of a combined approach of AV delay and VV interval optimization [25], [26].

The AV delay may be optimized with Doppler echocardiography by evaluating the aortic velocity time integral (AoVTI), which serves as a surrogate for LV stroke volume [18], [27], [28]. Three dimensional echocardiography (3DE) is an accurate and reproducible method to quantify LV dyssynchrony [29]. It is unclear if an elaborate echocardiographic approach to AV delay and VV interval optimization of CRT systems, including doppler echocardiography (AV delay optimization) and three-dimensional echocardiography (VV interval optimization), leads to an improved acute outcome after CRT initiation.

In the present study we therefore evaluated the feasibility of three-dimensional echocardiography (3DE) to optimize the inter-ventricular interval of biventricular pacemakers.

## Methods

### Ethics statement

All patients included in this study gave written informed consent prior to entry into the study. The study complies with the principles of the Declaration of Helsinki and was approved by the institutional ethics committee of Deutsches Herzzentrum München, Technical University of Munich, the only institution, where participants were recruited and this study was conducted (Az 2608/09; 10.12.2009).

### Study design

In this study we prospectively included patients who underwent CRT implantation according to 2006 guidelines [30] (ejection fraction <35%, New York Heart Association (NYHA) functional class III or IV despite optimal pharmacological therapy, and QRS duration >120 ms). At the time of inclusion baseline parameters were assessed (medication status, ECG, and echocardiography). All patients underwent a comprehensive evaluation with 3D echocardiography before implantation (baseline). The day after implantation the device was optimized as described below. Finally, LV function was again assessed echocardiographically.

### Echocardiography

**Figure 1:**
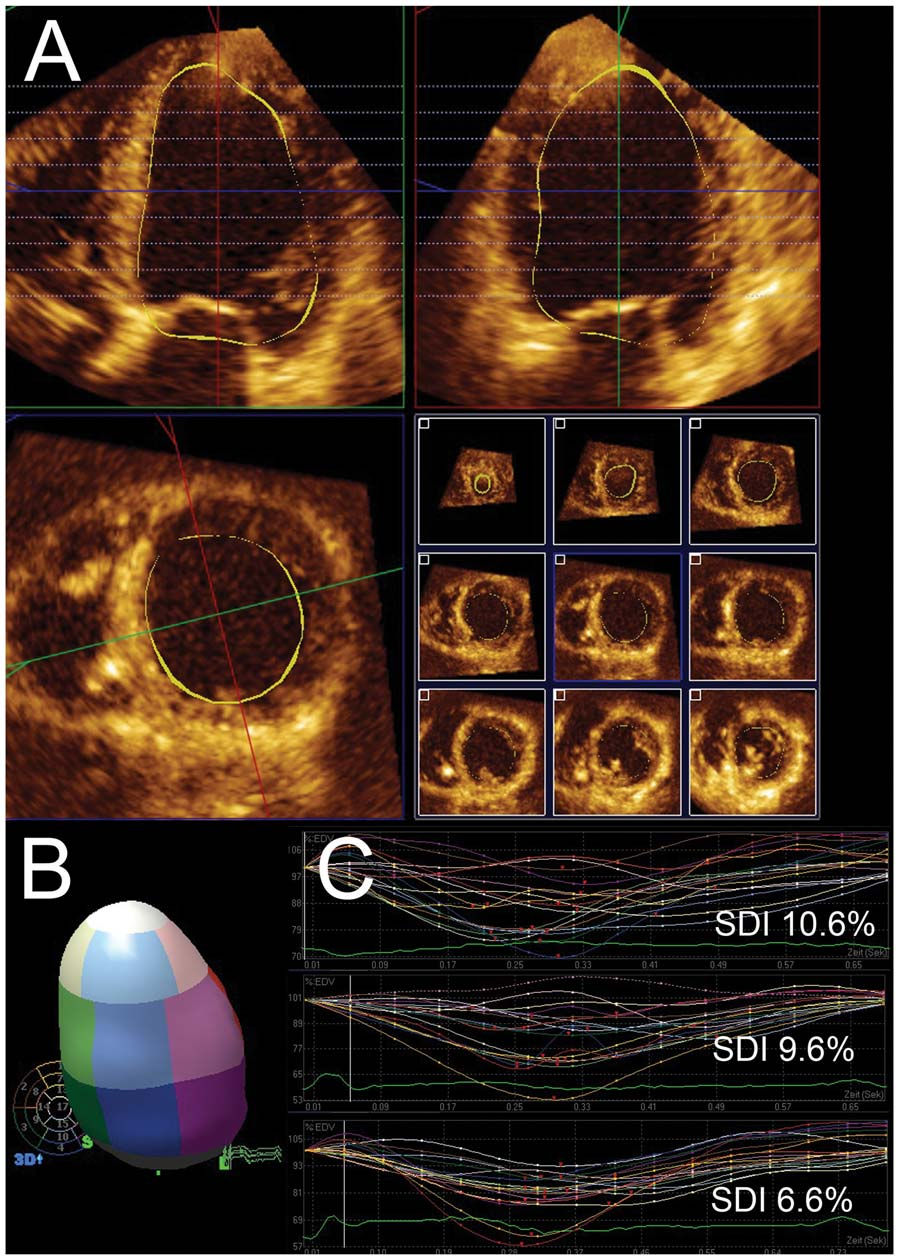
3D-echocardiography guided optimization of cardiac resynchronization therapy. Patient with ischemic cardiomyopathy, severly reduced systolic left ventricular (LV)-function (EF 16.8%), left bundle branch block and dyspnea on exertion (NYHA class III) selected for cardiac resynchronisation therapy (CRT) before biventricular pacemaker implantation: A) tracing of the LV endocardial boundary in the multiple apical and short axis views. From these datasets a volumetric model of the left ventricle is drawn (B), and time-volume curves are automatically integrated for each segment: C) time-volume curves of the patient before pacemaker implantation (upper panel); immediately after implantation (middle panel); after complete echocardiographic optimization (lower panel). Shown are the respective SDI values after each optimization step.

Routine baseline 2D and 3D echocardiography (Philips iE33) were performed in all patients according to institutional guidelines. 3D echocardiography was performed using the X3-1 matrix transducer. Apical views were optimized to allow complete visualization of the left ventricle in the 4- and 2-chamber views. During breath-hold a complete 3D volume was acquired during 7 to 8 cardiac cycles. Angle and depth were minimized to ensure optimal temporal resolution while still acquiring the entire LV volume. The volume data were sent to a workstation for off-line processing and analysis (QLab v. 6.0, Philips), using the 3DQ-Advanced plugin. The software allows semi-automated delineation of end-systolic and end-diastolic endocardial borders (Figure 1). Through automated sequential tracking throughout the cardiac cycle the software creates a dynamic 3D model of the left ventricular cavity. The software calculates ejection fraction, end-diastolic and end-systolic volumes, as well as the systolic dyssynchrony index (SDI). The latter is calculated as the standard deviation of the time to minimal systolic volume (TmSv) in 16 segments, excluding the apical cap in a standard 17-segment model. The SDI is corrected for the RR interval and is expressed as a percentage (Figure 1).

Echocardiographic guided optimization of the CRT device was performed the day after implantation. First, AV delays were analyzed from 80 to 200 ms, with steps of 20 ms. Aortic flow was recorded as a velocity-time integral (AoVTI) using continuous-wave Doppler at the level of the aortic valve, according to the consensus statement of the American Society of Echocardiography [31]. Subsequently, the AV delay with the highest velocity-time integral was programmed. Interventricular intervals between the right and left ventricles (VV intervals) were analyzed at 5 different intervals: simultaneous stimulation (LV = RV), left-ventricular pre-activation (LV +20, LV +40 ms), and right-ventricular pre-activation (RV +20, RV +40 ms). For each of the tested intervals a complete 3D full volume was acquired. Analysis of the 3D volume data was done off-line in a blinded fashion by 2 independent investigators. The device was programmed for the VV interval with the lowest SDI (Figure 1).

### Statistical analysis

All continuous variables are expressed as mean ± standard deviation. Presence of a normal distribution was evaluated by Kolmogorov-Smirnov test, means were compared by paired t-test. Where a non-parametric distribution was assumed, Wilcoxon test was performed. Categorical variables were tested by the chi-square test. To assess intra- and inter-observer variabilities, intra-class correlations (ICC) were calculated, and the variability is expressed as the absolute difference between 2 measurements divided by the average of the 2 measurements. Resulting p-values<0.05 were considered statistically significant.

## Results

### Baseline characteristics

A total of 25 patients were included in the study. The mean age was 67 years with 56% males (Table 1). 56% had ischemic cardiomyopathy as the underlying cause of heart failure. Further baseline characteristics are given in Table 1.

**Table 1:** Baseline characteristics.

	N = 25
Demographic data	
Age, years	67±11
Male sex	14 (56%)
**Clinical data**	
NYHA class 3	22 (88%)
NYHA class 4	3 (12%)
Ischemic CMP	14 (56%)
**Medication**	
Betablockers	23 (92%)
ACE-inhibitors/ARBs	23 (92%)
Diuretics	24 (96%)
Aldosterone antagonists	18 (72%)
Digitalis	3 (12%)
Statins	25 (100%)
**Electrocardiographic data**	
QRS width, ms (median; IQR)	160 (122–198)
**Echocardiographic data**	
LV end-diastolic volume, ml	176±62
LV end-systolic volume, ml	138±60
LVEF, %	23±7
SDI, %	14.3±5.5

Values are shown as means ± standard deviation or count (percentage).

NYHA, New York Heart Association; CMP, cardiomyopathy; ACE, Angiotensin-converting enzyme; ARB, Angiotensin receptor blocker; LV, left ventricle; SDI, systolic dyssynchrony index.

### AV delay optimization

**Figure 2:**
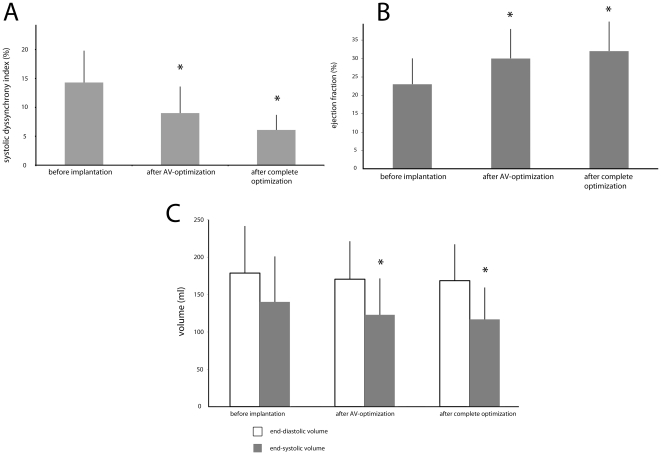
Acute hemodynamic effects of 3D-echocardiography guided optimization. Hemodynamic variables for each timestep of the optimization protocol: A) systolic dyssynchrony index, B) ejection fraction, and C) left-ventricular end-diastolic and end-systolic volumes. Shown are means ± standard deviation. * p<0.05 vs. baseline values.

For AV delay optimization aortic velocity time integral (AoVTI) was examined in eight different AV delays, and the AV delay with the highest AoVTI was programmed. Aortic flow increased from baseline 24±7 cm to 27±9 cm after AV delay optimization (p<0.05). The optimal mean sensed AV delay programmed was 108 ms [80–140 ms] and the optimal paced AV delay programmed was 133 ms [120–140 ms]. The SDI improved significantly from 14.3±5.5% to 9.0±4.6%, as well as the EF, which rose from a baseline value of 23±7% to 30±8% after AV delay optimization (Table 2, Figure 2).

**Table 2:** Echocardiographic parameters at baseline and after AV delay and VV interval optimization.

	Baseline	After AV delay optimization	After complete optimization
LV end-diastolic volume, ml	176±62	168±50	166±48
LV end-systolic volume, ml	138±60	121±48†	115±42¶ *
**Ejection fraction, %**	23±7	30±8†	32±8¶ *
**SDI, %**	14.3±5.5	9.0±4.†	6.1±2.6¶ *
**Aortic VTI, cm**	24±7	27±9†	26±8*

SDI, systolic dyssynchrony index; AV, atrio-ventricular; VV, ventriculo-ventricular; LV, left ventricular; VTI, velocity-time integral.

Shown are means ± standard deviation.

† p<0.001: for comparison of AV optimization vs baseline.

¶ p<0.05: for comparison of complete optimization vs AV optimization only.

*p<0.05: for comparison of complete optimization vs baseline.

### VV interval optimization

After AV delay optimization, the VV intervals were analyzed by evaluating the 3D full volume datasets. For each VV interval a separate 3D dataset was analyzed. The VV interval with the lowest corresponding SDI was used for programming the device.

We observed a further decrease in SDI values from 9.0±4.6% to 6.1±2.6% (Table 2). In most patients either simultaneous activation of left and right ventricle or mild pre-activation of the left ventricle resulted in the smallest SDI value (simultaneous activation in 28%, LV pre-activation by 20 ms in 48% of all patients). In 3 patients LV pre-activation by 40 ms, and in 3 patients right ventricular pre-activation by 20 ms led to the best SDI, and were programmed accordingly. Interestingly, the ejection fraction increased from 23±7% before pacemaker implantation to 30±8% after AV delay optimization and we could observe a further significant improvement after VV interval optimization (after complete optimization: 32±8%). The aortic VTI, which rose from baseline 24±7 to 27±9 cm after AV optimization, remained unchanged after VV interval optimization (26± cm). The end-diastolic volume fell from baseline 176±62 ml to 166±48 ml after complete optimization. The end-systolic volume also showed a significant reduction (baseline 138±60 ml, after complete optimization 115±42 ml; Table 2, Figure 2).

**Figure 3:**
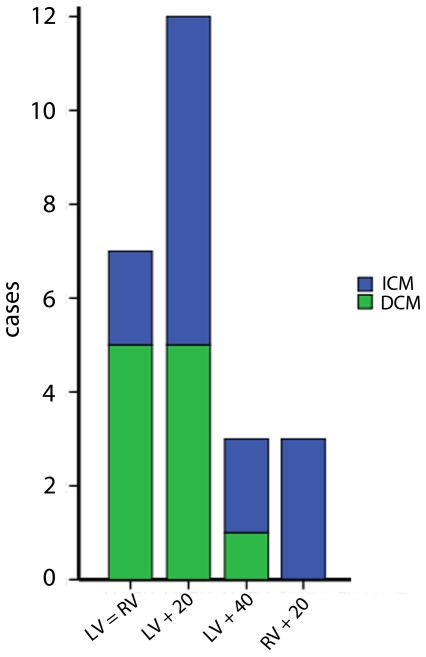
Ventricular-to-ventricular (VV) intervals in patients with ischemic and dilated cardiomyopathy. Number of patients with simultaneous activation of left and right ventricle or sequential inter-ventricular pacing depending on ischemic or dilated cardiomyopathy.

Only 28% of patients achieved the lowest possible SDI with the standard setting of simultaneous activation of left and right ventricle. 86% of patients with ischemic cardiomyopathy needed pre-activation versus only 55% of patients with dilated cardiomyopathy (p<0.05, Figure 3).

### Intra- and inter-observer agreement

The intra- and inter-observer agreement was generally very good.

Intra-observer variability in measured SDI was 14±8%. Intra-observer variability in ejection fraction, end-diastolic volume, and end-systolic volume was 3±2%, 2±2%, and 3±3%, respectively. Intra-class correlation coefficients were 0.974 for SDI, 0.997 for EF, 0.998 for EDV and 0.998 for ESV.

Inter-observer variability in measured SDI was 17±14%. Inter-observer variability in ejection fraction, end-diastolic volume, and end-systolic volume was 8±6%, 3±4%, and 4±3%, respectively. Intra-class correlation coefficients were 0.948 for SDI, 0.981 for EF, 0.997 for EDV and 0.996 for ESV.

## Discussion

In the present study we tested a new protocol of combined AV delay and VV interval optimization of CRT-systems including standard Doppler-echocardiography for AV delay, and 3D echocardiography for VV interval optimization. This elaborate protocol led to a significant improvement of LV function immediately after implantation of a CRT device compared to empiric device programming. The examined cohort existed of typical candidates for cardiac resynchronization therapy. All were highly symptomatic and on recommended optimal pharmacological therapy. Moreover, all patients showed QRS prolongation of more than 120 ms, and had a mean ejection fraction of 23%.

After the first step of optimization (AV delay optimization) the ejection fraction rose from 23% to 30% and could be increased even further by VV interval optimization (32%). End-systolic volume was reduced significantly already after AV delay optimization, with a moderate further reduction after VV interval optimization. The SDI, as a marker of interventricular dyssynchrony, fell sharply after each optimization step, whereas the aortic VTI, a surrogate marker of stroke volume, was only affected by AV delay optimization. In most patients the VV interval had to be programmed differently from the standard setting (i.e. simultaneous activation of left and right ventricle), only 28% of patients achieved the lowest possible SDI with the standard setting of simultaneous activation of left and right ventricle.

These results strongly support an individualized optimization of CRT-systems, specifically tailored to the patients. CRT is used in patients with several etiologies of severely symptomatic heart failure [9] [32], and one can imagine that a one fits all approach may not necessarily generate the best outcome. This has also been shown in a small cohort with Doppler optimization of AV delay and VV interval. Favorable hemodynamic response was more pronounced in the group of patients randomized to the optimization protocol [33].

This is the first study to use 3DE for VV interval optimization. The only modest further improvement of LV-EF after VV interval optimization in addition to AV optimization was lower than anticipated. Nevertheless, the pronounced decrease of the systolic dyssynchrony index (SDI) with each optimization step was striking and may lead to further improvement of LV-EF in the long-term. It is also known from a small study by Valzania et al. [34], that hemodynamic parameters tend to change over the course of several months after CRT. So, it would be very interesting, if these modest changes in EF immediately after implantation translate into robust changes later on in the course. Further follow-up has to clarify this issue.

The positive acute results are especially encouraging as LV resynchronization acutely after the implantation of biventricular pacemakers predicts response to CRT in the long-term [35]. Moreover a recent study by Kapetanakis et al. emphasized the relevance of the left ventricular systolic dyssynchrony index (SDI) with respect to patient selection for CRT [36]. They found SDI to be highly predictive of response to CRT, in terms of functional (NYHA functional class) and echocardiographic (LV-EF and LV end-systolic volume) improvement. Similarily, we found a trend towards more pronounced acute echocardiographic response with the suggested cutoff value for the baseline SDI of 10.4% (change in EF: 7 +/− 5% in the low SDI vs 10 +/− 4% in the high SDI subgroup, p = 0.1; change in ESV: −13 +/− 13 ml in the low vs. −26 +/− 30 ml in the high SDI subgroup, p = 0.2). Future studies should investigate if this highly promising optimization technique combined with improved selection criteria including an SDI cut-off would translate into improved functional outcome.

Furthermore, AV delay optimization can maximize the benefits of cardiac resynchronization therapy. If programmed poorly, it may curtail beneficial effects of VV interval optimization and of CRT in general. Optimized AV synchrony is achieved by an AV delay that provides the best left atrial contribution to LV filling resulting in maximum stroke volume. Several echocardiographic methods have been used for AV delay optimization [16], [17]. In our study, we used the aortic VTI Doppler method to optimize the AV delay. It produces reproducible results and has been shown to be superior to the mitral inflow method [37].

VV interval optimization has been attempted with several techniques with varying success [21], [22], [24], [25], [33], [38]. The high percentage of patients with sequential inter-ventricular pacing as the optimal setting is in concordance with other studies that evaluated VV interval optimization [22], [23], [24], [25], [39].

The SDI seems to be a promising parameter for VV interval optimization, as it may help to overcome the problems arising from LV-lead latency and from slowed conduction due to increased scar burden in patients with ischemic cardiomyopathy [13], [14]. Simultaneous pacing of both ventricles may result in suboptimal resynchronization due to an unbalanced activation of right ventricular and left ventricular wavefronts. SDI helps in finding and programming the optimal VV interval with either left or right ventricular pre-activation to compensate for these challenges and thus resulting in a more synchronous mechanical activation of both ventricles. Moreover, in a patient where the only possible LV-lead placement is in a presumed suboptimal location [40] pre-activation of either one of the ventricles may give the additional time to achieve synchronous mechanical activity. This applies predominantly to patients with ischemic cardiomyopathy where optimal lead placement might not be possible due to increased scarring in the area of the accessible veins. As has been previously shown by van Gelder et al. [39] we found that patients with ischemic cardiomyopathy needed more pre-activation than patients with dilated cardiomyopathy. 86% of patients with ischemic cardiomyopathy needed pre-activation versus only 55% of patients with dilated cardiomyopathy (p<0.05). Interestingly, patients with ischemic cardiomyopathy more often needed pre-activation of the right ventricle or extreme pre-activation of the left ventricle (>20 ms), whereas pts with dilated cardiomyopathy only needed slight to moderate pre-activation of the left ventricle (Figure 3) [41].

Although this optimization technique is more sophisticated and thus slightly more time consuming as compared to traditional echocardiographic optimization protocols due to offline analysis, it can be easily integrated into the usual workflow of post-operative CRT treatment in a patient with a hospital stay of 3 to 4 days. Images can be obtained postoperatively, AV delay programmed immediately and VV interval programmed after offline analysis before discharge from the hospital.

Several limitations apply to our study. This was a small study to evaluate a new concept of optimized resynchronization therapy. There was no control group in this study. Moreover no assumptions regarding the long-term benefit can be made. This has to be addressed in an adequately powered, prospective trial. The resolution of most 3D ultrasound scanners is still reduced compared to standard 2D technology. Especially, temporal resolution is still a major issue, leading to high variability of the measurements. This could hamper the analysis of small-scale variations of ventricular dyssynchrony and could thus influence the parameter setting with respect to the VV synchronization. Future technical improvements of 3D scanners might improve temporal as well as spatial resolution and lead to more reliable results.

### Conclusion

In the present study we could demonstrate, that an individualized echocardiographic optimization with the integration of 3-dimensional indices into the optimization protocol significantly improves LV function in CRT compared to empiric VV interval programming. This novel individualized echocardiographic optimization protocol can be used to select the optimal AV delay and VV interval in CRT.
